# Genetic Heterogeneity of the β-Globin Gene in Various Geographic Populations of Yunnan in Southwestern China

**DOI:** 10.1371/journal.pone.0122956

**Published:** 2015-04-07

**Authors:** Jie Zhang, Jing He, Xiao-Hong Zeng, Shi-Jun Ge, Yu Huang, Jie Su, Xue-Mei Ding, Ji-Qing Yang, Yong-Jiu Cao, Hong Chen, Ying-Hong Zhang, Bao-Sheng Zhu

**Affiliations:** 1 Genetic Diagnosis Center, Yunnan Provincial Key Laboratory For Birth Defects and Genetic Diseases, the First People’s Hospital of Yunnan Province, Kunming, People’s Republic of China; 2 Genetics Department, Kunming University of Science and Technology, Kunming, People’s Republic of China; 3 Department of Clinical Laboratory, the Peoples’ Hospital of Dehong Autonamic Prefecture of Dai and Jingpo, Mangshi, People’s Republic of China; 4 Department of Clinical Laboratory, Maternal and Child Health Hospital of Menghai, Menghai, People’s Republic of China; University of Naples Federico II, ITALY

## Abstract

**Objectives:**

The aim of this study was to investigate the geographic distribution of β-globin gene mutations in different ethnic groups in Yunnan province.

**Methods:**

From 2004 to 2014, 1,441 subjects with hemoglobin disorders, identified by PCR-reverse dot blot and DNA sequencing, were studied according to ethnicity and geographic origin. Haplotypes were examined among 41 unrelated thalassemia chromosomes.

**Results:**

Eighteen β-thalassemia mutations and seven hemoglobin variants were identified for 1,616 alleles in 22 different ethnic groups from all 16 prefecture-level divisions of Yunnan. The prevalence of β-thalassemia was heterogeneous and regionally specific. CD 41-42 (-TCTT) was the most prevalent mutation in the populations of northeastern Yunnan. CD 17 (A>T) was the most common mutation in the populations of southeastern Yunnan, especially for the Zhuang minority, whereas Hb E (CD 26, G>A) was the most prevalent mutation in populations of southwestern Yunnan, especially for the Dai minority. Among the seven types of haplotypes identified, CD 17 (A>T) was mainly linked to haplotype VII (+ - - - - - +) and IVS-II-654 (C>T) was only linked to haplotype I (+ - - - - + +).

**Conclusion:**

Our data underline the heterogeneity of β-globin gene mutations in Yunnan. This distribution of β-globin mutations in the geographic regions and ethnic populations provided a detailed ethnic basis and evolutionary view of humans in southern China, which will be beneficial for genetic counseling and prevention strategies.

## Introduction

β-thalassemia is one of the most common monogenic recessive diseases and it is the result of the presence of a mutation in one or two β-globin genes. It is characterized by decrease or absence of β-globin chain synthesis. β-thalassemia is prevalent in Mediterranean countries as well as in tropical Africa, southeast Asia and southern China. Approximately 800 different mutations of the β-globin gene have been documented in the HbVar database and more than 50 of them have been reported in China. The spectrum of β-globin gene mutations is variable within different ethnic groups. A specific racial group may have its own particular spectrum of mutations, reflecting significant genetic heterogeneity in the population. Characterizing the spectrum and frequency of β-globin gene mutations in different populations is useful for genetic counseling and prevention strategies.

The mutations responsible for β-thalassemia have been well documented in southern China, especially in Guangxi [1], Hongkong [2], Hainan [3] and Guangdong province [4]. However, mutations causing thalassemia have been studied less in the populations of Yunnan province. Yunnan province, a region located in southwestern China, has the Tropic of Cancer running through its southern part and has been known as the crucial gate of southeast Asia. The territory of Yunnan is largely tropical and consists of a subtropical plateau, especially in the southern and southwestern prefectures, and has the highest incidence of malaria in China [5]. It is characterized by a high incidence of hemoglobin variants [6]. Among the 56 recognized ethnic groups in China, twenty-six are found in Yunnan. The total population of Yunnan includes 46 million people and consists of many ethnic groups. Two-thirds are of the Han ethnic group (32 million), and the remaining population of 14 million consists of 25 different ethnic groups including Yi (11%), Hani (3.5%), Bai (3.4%), Dai (2.6%), Zhuang (2.6%) and others (national census in 2011).

Identification of β-globin gene mutations in the different regions will be helpful to explain the heterogeneity and prevalence of β-thalassemia in different ethnic regions. Moreover, the heterogeneity of these mutations can be used as genetic references to elucidate historical migrations and anthropological origins. The aim of this retrospective study, undertaken in the 16 prefecture-level divisions of Yunnan, was to determine the heterogeneity of thalassemia in different ethnic groups. To our knowledge, this is the first report on β-thalassemia mutations associated with the ethnic and regional variations in southwestern China. The results modify and expand the previously reported genetic information related to β-thalassemia in China.

## Materials and Methods

### Study Population and Detection of β-globin Mutations

From 2004 to 2014, a total of 1,441 individuals (aged 1–56 years) who were residents of Yunnan province were enrolled in the study. Hematological and clinical data associated with β-thalassemia obtained during genetic counseling, prenatal diagnosis and screening programs (programs conducted in the First Peoples’ Hospital of Yunnan Province, Peoples’ Hospital of Dehong, Maternal and Child Health Hospital of Wenshan, Maternal and Child Health Hospital of Xishuangbanna, Maternal and Child Health Hospital of Dehong, Maternal and Child Health Hospital of Lijiang) were analyzed. Blood samples were obtained for molecular analysis at either the First Peoples’ Hospital of Yunnan Province or the Peoples’ Hospital of Dehong Autonamic Prefecture of Dai and Jingpo. Clinical data were obtained from medical records of the patients and informed consent was signed by the participants or their guardians. The protocol and consent information for this study were approved by the Medical Ethics Committee of the First Peoples’ Hospital of Yunnan Province, PRC. Venous blood samples were collected in tubes with EDTA from hematological phenotype positive carriers and patients. A total of 17 β-globin mutations frequently seen in the Chinese population were detected by PCR-reverse dot blot (RDB) assays as previously described [7]. The entire β-globin gene, from the 5' promoter region to the 3' untranslated region (3' UTR), were sequenced in cases that did not have any of the 17 common mutations or in cases in which the genotype disagreed with the phenotype according to a previous report [7].

### Haplotype Analysis of the β-Globin Gene Cluster

Haplotypes throughout the β-globin gene cluster were determined by PCR-RFLP methodology and family linkage analysis as previously described [8]. The polymorphic sites were the following: 5′ to ε gene by Hind II, within IVS-II of the Gγ and Aγ genes by Hind III, within and 3′ to ψβ by Hind II, IVS-II of the β gene by Ava II and 3′ to the β gene by BamH I.

## Results

### β-Globin Mutations Identified in Yunnan

Eighteen β-globin gene mutations and seven hemoglobin variants were identified in 1,616 alleles included in the study (1,616 β-globin mutations in 2,882 alleles). Of the 1,441 subjects investigated, 175 were compound heterozygous or homozygous for β-globin mutations. Four rare β-globin mutations were detected for the first time in China; i.e., Hb Dieppe, CD 127 (A>G) (HBB:c.383A>G); initiation CD (T>C) (HBB:c.2T>C); Hb G-Copenhagen, CD 47 (G>A) (HBB:c.142G>A) and CD 5(-CT) (HBB:c.17_18delCT). The second cases of Hb G-Coushatta, CD 22 (A>C) (HBB:c.68A>C) [9] and CD 121 (G>T) (HBB:c.364G>T) [10] in mainland China were found in our study. The data were also analyzed according to five regions of Yunnan (Table 1 and Table 2), showing 406 (25.12%, 406/1616) alleles were found in the central region (Kunming, Yuxi, Chuxiong), 59 (3.65%, 59/1616) alleles were found in the northeast region (Zhaotong, Qujing), 63 (3.90%, 63/1616) alleles were found in the northwest region (Dali, Lijiang, Nujiang, Shangri-La), 815 (50.43%, 815/1616) alleles were found in the southwest region (Baoshan, Dehong, Xishuangbanna, Lincang, Puer) and 273 (16.90%, 273/1613) alleles were found in the southeast region (Wenshan, Honghe). The distribution pattern of β-globin gene mutations in 22 different ethnic groups (including one Khmer from Cambodia and four Shan from Myanmar) from all 16 prefecture-level divisions is also represented in Table 2 and Table 3. The frequency of Hb E (CD 26, G>A) (HBB:c.79G>A) was strikingly high in some minorities such as the Dai (60.13%, 353/587, Table 3) and CD 17 (A>T) (HBB:c.52A>T) was the most common mutation in the Zhuang minority (48.89%, 66/135, Table 3).

**Table 1:** Number of β-globin mutations found in five regions of Yunnan.

**Mutation**	**Type**	**Center**	**Northeast**	**Northwest**	**Southeast**	**Southwest**	**Total**
CD 26 (G>A) (HBB:c.79G>A)	β^+^	103	14	43	34	535	729 (45.1%)
CD 17 (A>T) (HBB:c.52A>T)	β^0^	100	17	3	107	110	337 (20.9%)
CD 41–42 (–TCTT) (HBB:c.126_129delCTTT)	β^0^	84	18	8	91	130	331 (20.5%)
IVS-II-654 (C>T) (HBB:c.316-197C>T)	β^+^	78	5	5	17	16	121 (7.49%)
CD 71/72 (+A) (HBB:c.216_217insA)	β^0^	8	1	-	7	16	32 (1.98%)
–28 (A>G) (HBB:c.-78A>C)	β^+^	11	1	1	12	6	31 (1.92%)
CD 27/28 (+C) (HBB:c.84_85insC)	β^0^	5	1	2	-	1	9 (0.56%)
IVS-I-1 (G>T) (HBB:c.92+1G>T)	β^0^	1	-	-	2	-	3 (0.19%)
CD 113 (T>A) (HBB:c.341T>A)	HbVar	3	-	-	-	-	3 (0.19%)
CD 121 (G>C) (HBB:c.364G>C)	HbVar	1	-	1	-	-	2 (0.12%)
IVS-I-5 (G>C) (HBB:c.92+5G>C)	β^+^	1	-	-	-	1	2 (0.12%)
-29 (A>G) (HBB:c.-79A>G)	β^+^	1	-	-	1	-	2 (0.12%)
CD 43 (G>T) (HBB:c.130G>T)	β^0^	-	1	-	1	-	2 (0.12%)
CD 56 (G>A) (HBB:c.170G>A)	HbVar	1	-	-	-	-	1 (0.06%)
IVS-I-2 (T>C) (HBB:c.92+2T>C)	β^0^	1	-	-	-	-	1 (0.06%)
–31 (A>C) (HBB:c.-81A>G)	β^+^	-	1	-	-	-	1 (0.06%)
CD 5 (–CT) (HBB:c.17_18delCT)	β^0^	-	-	-	1	-	1 (0.06%)
CD 22 (A>G) (HBB:c.68A>G)	HbVar	1	-	-	-	-	1 (0.06%)
CD 59 (G>C) (HBB:c.180G>C)	HbVar	1	-	-	-	-	1 (0.06%)
CD 121 (G>T) (HBB:c.364G>T)	β^0^	1	-	-	-	-	1 (0.06%)
CD 47 (G>A) (HBB:c.142G>A)	HbVar	1	-	-	-	-	1 (0.06%)
CD 22 (A>C) (HBB:c.68A>C)	HbVar	1	-	-	-	-	1 (0.06%)
CD 127 (A>G) (HBB:c.383A>G)	β^0^	1	-	-	-	-	1 (0.06%)
Initiation CD (T>C) (HBB:c.2T>C)	β^0^	1	-	-	-	-	1 (0.06%)
CAP +40 to +43 (-AAAC) (HBB:c.-11_-8delAAAC)	β^+^	1	-	-	-	-	1 (0.06%)
Total		406 (25.12%)	59 (3.65%)	63 (3.90%)	273 (18.9%)	815 (50.4%)	1616 (100%)

β^0^: production of β-globin chain is entirely eliminated; β^+^: production of β-globin chain is reduced; HbVar: hemoglobin variant.

**Table 2:** Number of alleles form 22 different ethnic groups in five regions of Yunnan.

**Ethnicity**	**Center**	**Northeast**	**Northwest**	**Southeast**	**Southwest**	**Total**
Han	336	51	27	91	129	634(39.2%)
Dai	10	-	-	3	574	587(36.3%)
Zhuang	10	-	-	125	-	135(8.35%)
Yi	22	2	4	33	5	66(4.08%)
Bai	2	-	20	-	5	27(1.67%)
Achang	-	-	-	-	27	27(1.67%)
Jingpo	-	-	-	1	26	27(1.67%)
Lisu	2	-	4	-	15	21(1.30%)
Hui	9	2	3	2	2	18(1.11%)
Miao	4	1	-	8	-	13(0.80%)
Deang	-	-	-	-	13	13(0.80%)
Hani	1	-	-	2	7	10(0.62%)
Buyi	5	2	-	1	1	9(0.56%)
Yao	-	-	-	7	1	8(0.50%)
Naxi	2	-	5	-	-	7(0.43%)
Shan	1	-	-	-	4	5(0.25%)
Jinuo	-	-	-	-	3	3(0.19%)
Wa	-	-	-	-	2	2(0.12%)
Shui	-	1	-	-	-	1(0.06%)
Lafu	-	-	-	-	1	1(0.06%)
Khmer	1	-	-	-	-	1(0.06%)
Dong	1	-	-	-	-	1(0.06%)
Total	406	59	63	273	815	1616

One Khmer come from Cambodia, lived in Kunming; Four Shan come from Myanmar, one lived in Kunming and three lived in Dehong.

**Table 3:** The frequency of β-thalassemia mutations in different ethnic populations.

Minority	CD 26 (G>A)	CD 17 (A>T)	CD 41–42 (–TCTT)	IVS-II-654 (C>T)	CD 71/72 (+A)	–28 (A>G)	Others^A^	Total
Han	200	142	137	97	11	17	30	634
Dai	353	88	119	7	14	5	1	587
Zhuang	3	66	53	2	6	3	2	135
Yi	34	14	4	5	1	6	2	66
Bai	20	3	4	-	-	-	-	27
Achang	27	-	-	-	-	-	-	27
Jingpo	27	-	-	-	-	-	-	27
Lisu	20	1	-	-	-	-	-	21
Hui	9	5	1	3	-	-	-	18
Miao	-	10	2	1	-	-	-	13
Deang	13	-	-	-	-	-	-	13
Others^B^	23	8	11	6	-	-	-	48
Total	729	337	331	121	32	31	35	1616

^A^: other 12 β-thalassemia mutations and 7 hemoglobin variants mutations;

^B^: other minority ethnic groups, including Hani, Yao, Buyi, Naxi, Dong, Shui, Wa, Jinuo, Lafu, Khmer and Shan.

### Prevalence of β-Thalassemia in the Center of Yunnan

**Fig 1:**
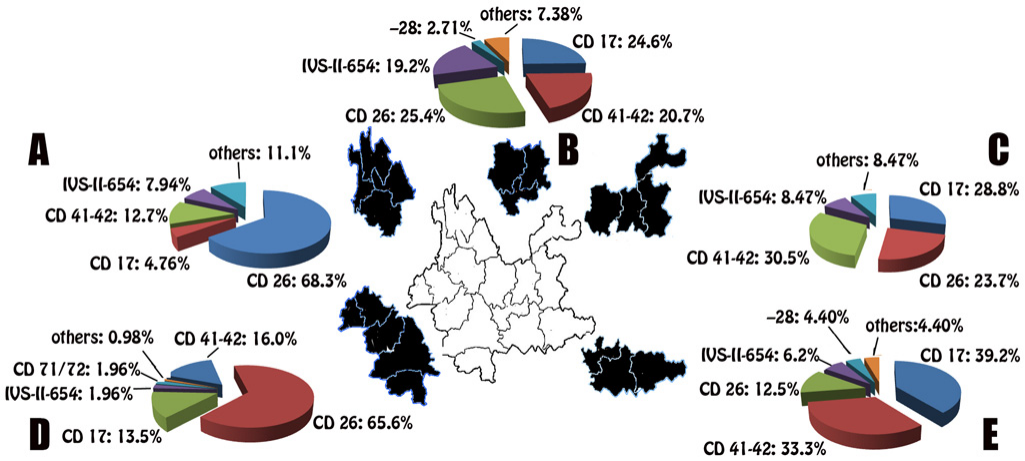
Geographic distribution of β-thalassemia mutations in five regions of Yunnan. A: northwester Yunnan; B: central Yunnan; C: northeastern Yunnan; D: southwestern Yunnan; E: southeastern Yunnan.

In the center of Yunnan, Hb E (CD 26, G>A) (HBB:c.79G>A) (25.4%, 103/406), CD 17 (A>T) (HBB:c.52A>T) (24.63%, 100/406), CD 41–42 (-TCTT) (HBB:c.126_129delCTTT) (20.69%, 84/406) and IVS-II-654 (C>T) (HBB:c.316-197C>T) (19.21%, 78/406) mutations were the most frequent (Table 1 and Fig 1). The majority of β-globin gene mutations screened in this region were found in the Han ethnic group (82.76%, 336/406) while 5.42% were in the Yi ethnic group (22/406), 2.46% (10/406) were in the Zhuang ethnic group and 2.22% (9/406) were in the Muslim (Hui) ethnic group (Table 2). Direct sequencing identified a number of rare mutations, including Hb Dieppe, CD 127 (A>G) (HBB:c.383A>G); initiation CD (T>C) (HBB:c.2T>C); Hb G-Copenhagen, CD 47 (G>A) (HBB:c.142G>A); Hb G-Coushatta, CD 22 (A>C) (HBB:c.68A>C); CD 121 (G>T) (HBB:c.364G>T); Hb D-Los Angeles, CD121 (G>C) (HBB:c.364G>C); Hb G-Taipei, CD 22 (A>G) (HBB:c.68A>G); Hb New York, CD 113 (T>A) (HBB:c.341T>A); Hb J-Bangkok, CD 56 (G>A) (HBB:c.170G>A); Hb J-Lome, CD 59 (G>C) (HBB:c.180G>C) and IVS-I-2 (T>C) (HBB:c.92+2T>C) (Table 1).

### Prevalence of β-Thalassemia in Southern Yunnan

Analysis of 815 alleles from the southwestern region showed that the most common mutations were Hb E (CD 26, G>A) (HBB:c.79G>A) (65.64%, 535/815), CD 41–42 (-TCTT) (HBB:c.126_129delCTTT) (15.95%, 130/815), CD 17 (A>T) (HBB:c.52A>T) (13.50%, 110/815), CD 71–72 (+A) (HBB:c.216_217insA) (1.96%, 16/815) and IVS-II-654 (C>T) (HBB:c.316-197C>T) (1.96%, 16/815), which were found in 99.02% (807/815) of the β-globin genes in this region (Table 1 and Fig 1). Among the patients screened in this area, 157 were β-thalassemia/Hb E or Hb E homozygous. Chromosomes screened in this area were mainly from the Dai ethnic group (70.43%, 574/815) and the remainder were from the 11 different ethnic groups (15.83% Han, 3.31% Achang, 3.19% Jingpo, 1.84% Lisu and others; Table 2). The Hb E (CD 26, G>A) mutation was highly prevalent in almost all of the southwestern populations that were screened and was especially prevalent in Achang (100%, 27/27), Jingpo (100%, 26/26) and Deang (100%, 13/13; Table 3).

In the southeast region, the most common mutations were CD 17 (A>T) (HBB:c.52A>T) (39.19%, 107/273), followed by CD 41–42 (-TCTT) (HBB:c.126_129delCTTT) (33.33%, 91/273), Hb E (CD 26, G>A) (HBB:c.79G>A) (12.45%, 34/273) and IVS-II-654 (C>T) (HBB:c.316-197C>T) (6.23%, 17/273) (Table 1 and Fig 1). Six other mutations were identified and all were at a frequency of less than 9%. Most of the alleles screened were found in the minority populations, including the Zhuang (45.79%, 125/273), Yi (12.09%, 33/273), Miao (2.93%, 8/273) and others.

### Prevalence of β-Thalassemia in Northern Yunnan

In the northeast region, the most common mutations were CD 41–42 (-TCTT) (HBB:c.126_129delCTTT) (30.50%, 18/59) and CD 17 (A>T) (HBB:c.52A>T) (28.81%, 17/59). In the northwest region, the most common mutations were Hb E (CD 26, G>A) (HBB:c.79G>A) (68.25%, 43/63), CD 41–42 (-TCTT) (HBB:c.126_129delCTTT) (12.70%, 8/63), IVS-II-654 (C>T) (HBB:c.316-197C>T) (7.94%, 5/63) and CD 17 (A>T) (HBB:c.52A>T) (4.76%, 3/63; Table 1 and Fig 1).

### β-Thalassemia Mutation Associations in Haplotypes

Haplotype analysis for 41 unrelated thalassemia chromosomes associated with seven mutations were analyzed (Table 4). Seven different haplotypes were identified including haplotype I (9), haplotype III (4), haplotype IV (2), haplotype V (3), haplotype VII (19),- - - - - + + (atypical haplotype, 3) and - - + - - - + (atypical haplotype, 1).

**Table 4:** β-thalassemia mutations and their haplotypes in the Yunnan population.

**Mutation**	**Origin and Minority**	**Haplotype**
CD 41–42 (–TCTT)	Xishuangbanna, Dai (3); Wenshan, Han (2); Kunming, Han (1)	+ - - - - - + (VII)
	Xishuangbanna, Dai (1)	+ - - - - + - (V)
	Xishuangbanna, Dai (3)	- - - - - + +^A^
	Qujing, Han (1), Wenshan, Yao (2); Chuxiong, Han (1); Xishuangbanna, Dai (1)	+ - - - - + + (I)
CD 17 (A>T)	Chuxiong, Han (1); Yuxi, Dai (2); Kunming, Han (2); Kunming, Zhuang (1); Dali,Yi (1); Zhaotong, Han (1); Xishuangbanna, Dai (2)	+ - - - - - + (VII)
	Wenshan, Han (1)	- + - + + - + (IV)
CD 26 (G>A)	Chuxiong, Han (1); Dehong, Dai (2); Xishuangbanna, Dai (1)	- + - + + + - (III)
	Puer, Han (2)	+ - - - - + - (V)
CD 47 (G>A)	Kunming, Han (1)	- - + - - - +^B^
IVS-II-654 (C>T)	Kunming, Buyi (1); Kunming, Han (1); Chuxiong, Han (1); Wenshan, Yi(1)	+ - - - - + + (I)
IVS-I-1 (G>T)	Wenshan, Han (1)	+ - - - - - + (VII)
CD 71/72 (+A)	Kunming, Han (1); Yuxi, Dai (1)	+ - - - - - + (VII)
CD 27/28 (+C)	Xishuangbanna, Han (1)	- + - + + - + (IV)

A and B: atypical haplotype.

## Discussion

This is the first study on β-thalassemia carriers and other hemoglobinopathies covering 22 different ethnic groups from different regions of Yunnan. Knowledge of the geographic and ethnic distributions of β-thalassemia mutations has useful implications for the prevention and control of thalassemia in China. The data indicated that there were marked differences in the geographical distributions of β-thalassemia mutations within different racial groups, highlighting the ethnic diversity in Yunnan. Hb E (CD 26, G>A) (65.64%) and CD 41–42 (-TCTT) (30.5%) were the most prevalent mutations in the southwest and northeast regions, respectively, whereas CD 17 (A>T) (39.19%) was the most common mutation in the southeastern Yunnan population.

In the southeastern region, in which Zhuang is the largest group (45.79%), CD 17 (A>T) (39.19%) was the most common mutation followed by CD 41–42 (-TCTT) (33.33%). This situation was similar to the frequency of β-thalassemia in the neighboring Guangxi Zhuang autonomous region, where the most common mutation was CD 41–42 (-TCTT) (39.4%) and CD 17 (A>T) (32%) [11]. Hb E (CD 26, G>A) (65.64%) and CD 41–42 (-TCTT) (15.95%) were the most prevalent mutations in the southwestern region, especially for the Dai minority group (70.43%), which was similar to the Thai population in Thailand [12–14]. The Thai population includes immigrants of the ancestors of the present-day Dai people from Yunnan [15].

In the southwest region, Hb E (CD 26, G>A) (65.64%) was the most common mutation observed in the local minority groups, especially for the native people who live in the tropical plateau; i.e., Achang (100%), Jingpo (100%) and Deang (100%), which has not been reported in other populations in China. The high frequency of Hb E (CD 26, G>A) in this region was similar to that of adjacent countries, such as Myanmar [16], Thailand and Laos [13], where a “hot spot” of Hb E (CD 26, G>A) is located on the Thai-Laos-Cambodia border [17]. A plausible explanation for the high incidence of Hb E (CD 26, G>A) was that carriers of Hb E (CD 26, G>A) were protected against falciparum malaria [18]. The geographical distribution of Hb E (CD 26, G>A) in Yunnan co-exists with the distribution of previous malaria epidemics [19], suggesting that the high frequency of Hb E might be associated with natural selective inhibition of malaria [20]. Secondly, it may be due to high consanguinity in minority groups with very restricted population movement caused by sociocultural factors and difficult travel potential associated with being surrounded by large mountains, which increases the risks of β-thalassemia/Hb E compound heterozygosity and Hb E homozygosity.

In the center region, where many different ethnic groups (14 ethnic groups in this study) reside, representing nearly 12 million habitants, genetic heterogeneity has led to a admixture of different β-thalassemia mutations. A total of 22 β-globin mutations, including 3 mutations that have not been reported previously in China, were detected in the 406 alleles, which accounted for two-fifths of the β-thalassemia mutations reported in China. Hb E (CD 26, G>A) (25.4%) and CD 17 (A>T) (24.6%) were the most common mutations. Among the mutations that were identified, CD 127 (A>G) was a very rare dominant β-thalassemia mutation first discovered in a 31-year-old French female 21 years ago [21]. Initiation CD (T>C) was observed in Yugoslavia in 1990 [22], but never in China. CD 47 (G>A) was a rare mutation detected in Danish, Sicilian and families with African ancestry [23]. The heterogeneity of β-thalassemia in this region might be due to the fact that the center of Yunnan, as a capital ruled by Yi, Mongols and Han people throughout history, is a crucial strategic crossroad between China and southern Asia. Also, the Japanese invasion (1940 AD) forced various immigrants into this region. Centuries of influx of many people resulted in great heterogeneity in the population. It has been suggested that the common mutations may be indigenous to the native population of Yunnan, whereas the rare β-globin mutations may have been introduced by immigration.

In the present study, seven types of haplotypes were found in 41 β-thalassemic chromosomes. Haplotype VII (+ - - - - - +, 19 chromosomes) and haplotype I (+ - - - - + +, 9 chromosomes) were the most prevalent haplotypes among β-thalassemia patients. The findings revealed that the haplotype VII (+ - - - - - +) accounted for 90.9% (10/11) of the CD 17 (A>T) alleles examined. This linkage was similar to the results of surveys carried out in India [24], Korean [25] and South Vietnam [26]. IVS-II-654 (C>T) was linked to only one type of haplotype: + - - - - + + (haplotype I) and haplotype I in association with IVS-II-654 (C>T) has been previously reported in China [6] and Indonesia [27].

The mutation CD 41–42 (-TCTT) was associated mainly with + - - - - - + (haplotype VII) and + - - - - + + (haplotype I), which had been described earlier in southern China [28–29]. The haplotype background associated with Hb E (CD 26, G>A) was mainly - + - + + + - (haplotype III), which was similar to that found in patients from the eastern region of India [30]. It was identified for the first time that Hb G-Copenhagen (CD 47, G>A) was associated with the haplotype - - + - - - +, a rare haplotype seldomly reported in β-thalassemia patients in China. The remaining patients had haplotype IV, haplotype V and - - - - - + + (atypical haplotype).

Our study has shown the remarkable diversity of β-thalassemia in different geographic regions in Yunnan. This information on the epidemiology of hemoglobin disorders could be helpful for planning appropriate treatments for β-thalassemia patients according to different population groups. Until now, there have been few studies regarding β-thalassemia carriers in minority groups and the evolution of haplotypes of the β-hemoglobin gene. Our study provided useful information to pave the way for prenatal diagnosis programs and large-scale population screening for β-thalassemia in Yunnan.
